# Assessing the transferability of micro-computed tomography (CT)-based plaque segmentation to clinical CT using super-resolution: Limits and emerging perspectives

**DOI:** 10.1016/j.jvssci.2026.100429

**Published:** 2026-06-20

**Authors:** Salomé H. Kuntz, Katherine Morales, Hugo Gangloff, Yohan Petetin, Anne Lejay, Nabil Chakfé

**Affiliations:** a Gepromed, Strasbourg, France; b Department of Vascular Surgery, Kidney Transplantation and Innovation, Strasbourg University Hospitals, Strasbourg, France; c UR 3072, Mitochondrie Stress Oxydant et Plasticité Musculaire, Strasbourg, France; d Telecom SudParis, Evry, France; e Université Paris Saclay, AgroParis Tech, INRAE, UMR MIA, Paris, France

**Keywords:** Artificial intelligence, Atherosclerotic vascular disease, Computed tomography angiography, Diagnostic imaging, Peripheral arterial disease

## Abstract

Noninvasive plaque characterization remains limited in peripheral arterial obstructive disease (PAOD), whereas ex vivo micro-computed tomography (micro-CT) enables quasihistological assessment. This study evaluates whether plaque-related structural information—defined as compositional and morphological plaque features learned from micro-CT segmentation—can be transferred to clinical computed tomography (CT) using a super-resolution (SR) framework. Popliteal artery segments from six patients with peripheral arterial obstructive disease were analyzed using micro-CT and histology. Annotated micro-CT images were used to train convolutional neural networks for plaque segmentation. Low-resolution clinical CT images were upsampled using a Laplacian pyramid SR approach, and segmentation models were applied without retraining. Performance was assessed using Dice scores on held-out micro-CT test data, and segmentation outputs on SR-CT images were qualitatively evaluated. Segmentation of calcified plaque components on micro-CT test images yielded Dice scores ranging from 0.58 to 0.67, indicating low-to-intermediate agreement. When applied to SR-CT images, segmentation revealed nonrandom identification of calcified structures in selected image sequences, with marked heterogeneity across slices. SR enables exploratory assessment of plaque information transfer but does not overcome the fundamental resolution gap. These findings define the current limits of CT-based plaque characterization and provide a framework for evaluating future imaging technologies.

**Clinical Relevance:**

Accurate plaque characterization remains a major unmet need in peripheral arterial disease, where treatment planning is largely guided by lesion length and stenosis severity rather than plaque composition. This study proposes using micro-computed tomography (micro-CT) as a histology-informed reference to evaluate how much plaque-related information can be transferred to clinical CT. By explicitly defining current limitations, our findings caution against premature clinical application while informing future developments in advanced CT technologies, multimodal imaging, and artificial intelligence.

Peripheral arterial obstructive disease (PAOD) is associated with substantial morbidity and risk of limb loss.^1^ Current treatment planning relies largely on lesion length and degree of stenosis assessed on computed tomography (CT), whereas plaque composition—known to influence procedural complexity and healing—remains poorly characterized noninvasively. Histological studies and ex vivo imaging have demonstrated marked heterogeneity of peripheral atherosclerotic plaques, particularly regarding calcification patterns, which may impact endovascular device performance.^2^^,^^3^

We demonstrated that micro-computed tomography (micro-CT) enables high-resolution, quasihistological assessment of atherosclerotic plaques ex vivo, and that it has been successfully combined with deep learning for automated plaque segmentation.^4^^,^^5^ However, the resolution gap between micro-CT and routine clinical CT precludes direct translation. Even high-resolution CT and emerging photon-counting CT remain far below micro-CT spatial resolution.^6^

Recent machine learning approaches have attempted PAOD characterization on CT^7^^,^^8^; however, these methods are limited by the absence of histological ground truth and by the intrinsic spatial resolution constraints of clinical CT.^9^ Although calcified components can be reliably detected, differentiation of noncalcified plaque remains challenging, limiting biological interpretability and clinical applicability.

Rather than proposing a new segmentation architecture or claiming clinical applicability, this study explores whether super-resolution (SR) may serve as a computational intermediary to test the transferability of plaque segmentation learned from micro-CT to clinical CT images. By explicitly evaluating both the potential and the limitations of this approach, we aim to define a framework for future translational development.

## Methods

### Specimens and imaging

**Fig 1:**
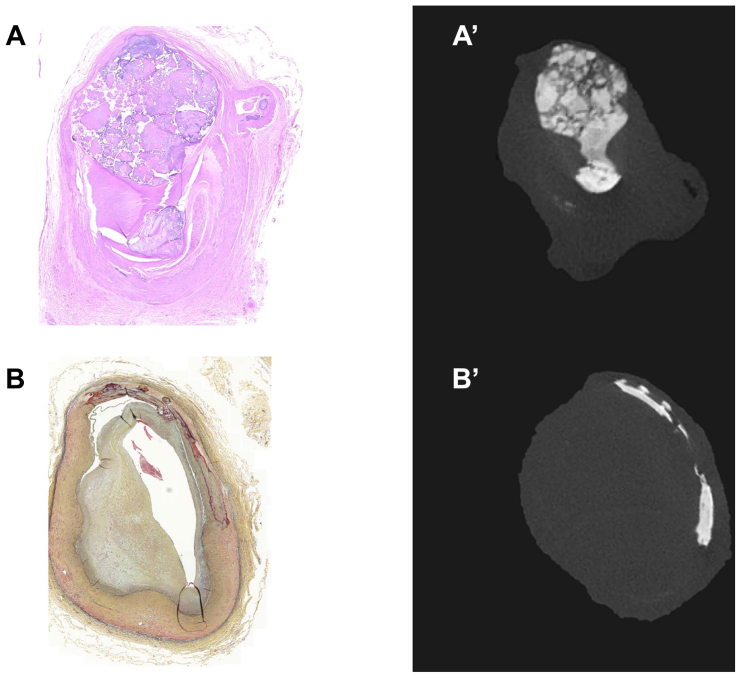
Example of coregistration between histological sections and their micro-computed tomography (CT) coregistrations. **A** and **B,** Histological cross-sections, and **A′** and **B′,** show corresponding micro-CT coregistrations.

Popliteal artery segments were harvested from six patients undergoing above-knee amputation for advanced PAOD, with informed consent for the study and publication. This study was approved by an ethics committee at the University Hospital of Strasbourg (2018-A03406-49 RIPH3). Specimens were imaged using micro-CT [Nikon X-Tek XT H 225ST Micro-CT system (Nikon Metrology) with a PerkinElmer PE1621 EHS 2000 × 2000 X ray detector panel (PerkinElmer)] and processed for histology using Movat pentachrome and hematoxylin-eosin staining. Micro-CT images were coregistered with histological sections using anatomical landmarks and calcification patterns (Fig 1). Clinical CT data were obtained from standard preoperative CT angiography (a 320-row scanner; Aquilion One, Canon Medical Systems) with inframillimetric reconstructions). Low-resolution CT cross-sections corresponding to the arterial segments were extracted for computational analysis. Coregistration between micro-CT and histology was performed using anatomical landmarks and calcification patterns. The exact method of coregistration has already been published.^4^

### Annotation and classes

A total of 91 coregistered cross-sections were obtained, including 81 used for training and 10 for testing. Slices were distributed across six patients, with multiple slices per specimen. Owing to the limited sample size and spatial correlation between slices, a patient-level split and a separate validation set were not feasible. Test data were strictly excluded from training.

Micro-CT cross-sections were manually annotated by an expert in plaque histopathology, using corresponding histological slides as reference. Two segmentation configurations were explored: (1) three classes (such as background, nodular calcification, and sheet calcification), focusing on reliably detectable calcified components; and (2) four classes (such as background, soft tissue, nodular calcification, and sheet calcification); to assess whether more complex biological information could be captured despite known CT limitations.

### Super-resolution

**Fig 2:**
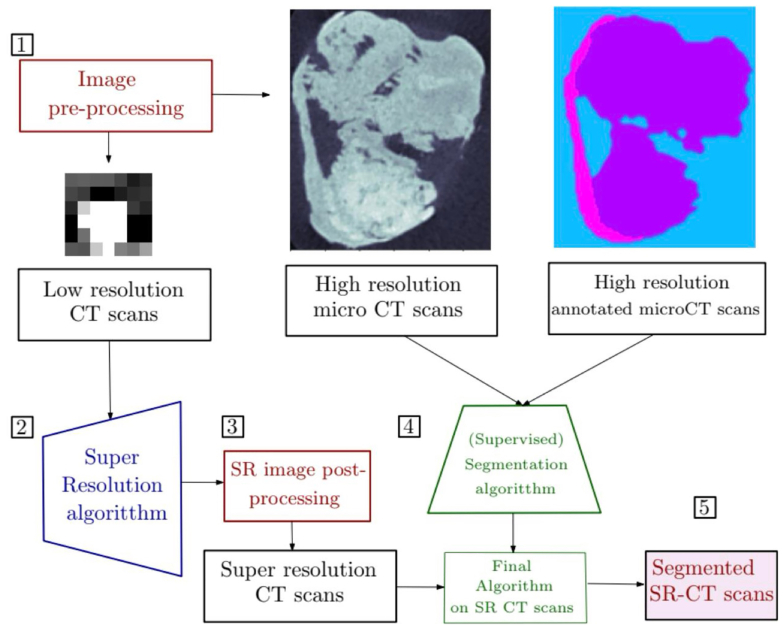
Our workflow for segmenting sheet and nodular calcifications in computed tomography (CT) images of the femoropopliteal segment is structured into five steps. First, we perform preprocessing of the CT and micro-CT images, followed by a super-resolution (SR) algorithm to the CT images, postprocessing of the SR-CT images, supervised segmentation, and segmentation on the SR-CT images.

**Fig 3:**
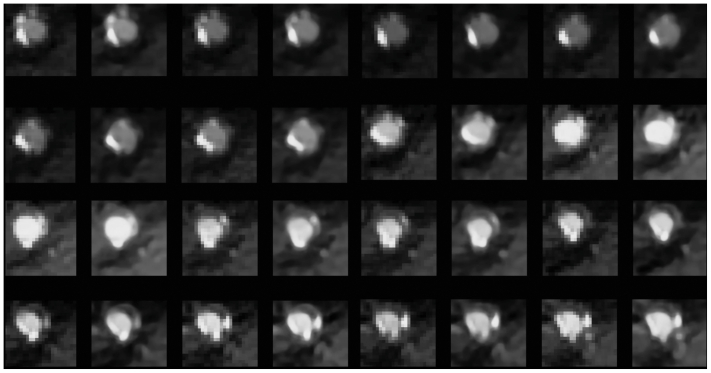
Example of a sequence of computed tomography (CT) and super-resolution (SR)-CT images. From *left* to *right*, the pairs of images [low-resolution (LR) CT, reconstructed SR-CT). The CT images correspond to a sequence of two-dimensional (2D) slices of a three-dimensional (3D) CT image, where the calcifications are present. The corresponding ST-CT images are obtained with the LapSRN algorithm with a factor of upscaling of eight.

Low-resolution CT images (12 × 12 pixels) were upsampled to 96 × 96 pixels using a Laplacian Pyramid Super-Resolution Network. Intensity normalization was applied to reduce distributional mismatch with micro-CT images.^10^ SR images were considered computational representations rather than anatomically accurate reconstructions (Figs 2 and 3).

### Segmentation models

**Fig 4:**
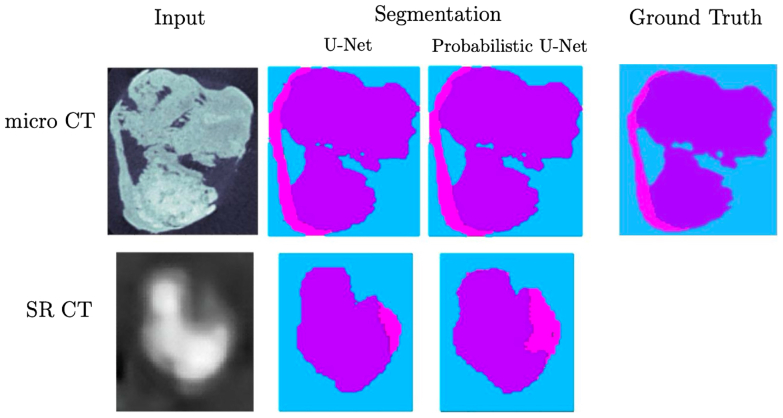
Example of a segmentation of the micro-computed tomography (CT) (first row) and its corresponding super-resolution-CT images (second row) with the U-Net and Probabilistic U-Net models (3 classes).

Segmentation models were trained exclusively on annotated micro-CT images. A standard U-Net architecture was used to assess the presence of transferable information rather than to optimize segmentation performance, as illustrated in Fig 4. A Probabilistic U-Net was evaluated in an exploratory manner to account for segmentation ambiguity.^11^ Models were evaluated on held-out micro-CT test images using Dice scores and subsequently applied to SR-CT images without further optimization.

Due to the small sample size and nonindependence of slices, no formal statistical comparison between models was performed.

## Results

Six patients (5 males, 1 female) with a median age of 69.9 years (interquartile range, 59.6-84.1 years) had undergone above-the-knee-amputation; yielding 91 coregistered histological and micro-CT cross-sections.

For three-class segmentation, Dice scores were 0.77 for the background, 0.60 for nodular calcifications, and 0.59 for sheet calcifications with the U-Net model. Dice scores were 0.72 for the background, 0.62 for nodular calcifications, and 0.61 for sheet calcifications with the Probabilistic U-Net model.

For four class-segmentation, Dice scores were 0.60 for the background, 0.63 for the soft tissue, 0.58 for nodular calcifications, and 0.60 for sheet calcifications with the U-Net model. Dice scores were 0.62 for the background, 0.64 for the soft tissue, 0.58 for nodular calcifications, and 0.67 for sheet calcifications with the Probabilistic U-Net model.

**Fig 5:**
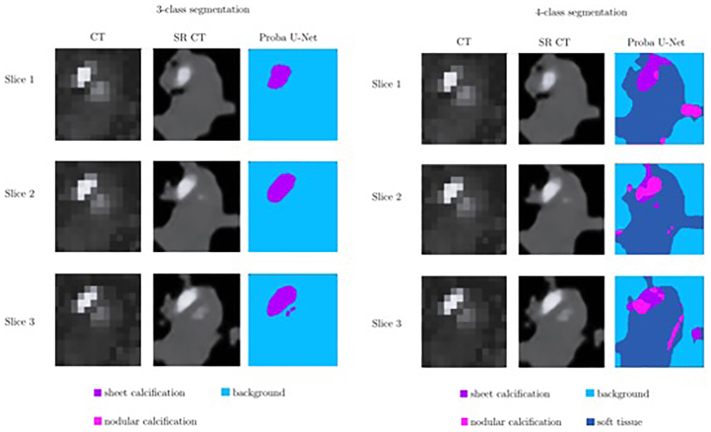
The three-class model: Three consecutive super-resolution (SR) (×8 LapSRN) computed tomography (CT) slices and their segmentations produced by the Probabilistic U-Net model, under three-class (such as background, nodular calcification, and sheet calcification). The consecutive nature of the slices demonstrates the spatial consistency of the segmentation: the predicted regions evolve smoothly across slices, confirming that the model captures meaningful anatomical structure rather than producing random outputs. The four-class model: Three consecutive SR-CT slices and their segmentations produced by the Probabilistic U-Net in the four-class configuration (such as background, soft tissue, nodular calcification, and sheet calcification). Adding the soft-tissue label highlights the network’s capacity to distinguish noncalcified plaque; the smooth evolution of all four regions across slices attests to spatial consistency.

These values indicate limited but nonrandom segmentation performance in a challenging multiclass setting. Fig 5 presents the results obtained with the Probabilistic U-Net model, which achieved the highest Dice scores. Although the standard U-Net yielded slightly lower quantitative performance, the visual appearance of the resulting segmentations was very similar, with no major qualitative differences observed. For this reason, only the Probabilistic U-Net results are displayed.

When applied to SR-CT images, both models were able to identify calcified plaque components in selected image sequences. Segmentation quality was heterogeneous and required expert interpretation, reflecting the residual gap between SR-CT and micro-CT image domains. No formal statistical comparison between models was performed.

## Discussion

In this study, we explored whether SR could act as a computational intermediary to assess the transferability of plaque-related information learned from ex vivo micro-CT to clinical CT. Rather than proposing a clinically deployable solution, our objective was to quantify the extent to which structural and compositional plaque features learned from a histology-informed, high-resolution reference domain may be inferred from routine CT imaging.

Our results show that calcified plaque components can be identified in SR-CT images in a nonrandom manner, albeit with low-to-intermediate agreement metrics. Dice scores below 0.7 reflect both the intrinsic complexity of plaque segmentation and the substantial resolution and domain gap between micro-CT and clinical CT. Importantly, these findings indicate that the primary limitation is not algorithmic but structural, reflecting the fundamental mismatch between the information content of micro-CT and clinical CT. Increasing model complexity, including probabilistic approaches, did not substantially improve performance, supporting this interpretation.

The inclusion of a soft tissue class further highlights these limitations. CT—and to some extent micro-CT—has limited contrast resolution for noncalcified components, which likely explains the reduced performance observed in the four-class configuration. This limitation is clinically relevant, as differentiation between fibrotic and softer plaque components remains a key challenge in endovascular planning.

From a methodological standpoint, our results also illustrate the inherent challenges of deep learning-based plaque segmentation. Class imbalance, overlapping intensity distributions, and gradual transitions between tissue types contribute to segmentation uncertainty. Although data augmentation strategies partially mitigate class imbalance, they do not resolve the fundamental similarity between plaque components. The use of a Probabilistic U-Net was motivated by this intrinsic ambiguity. By modelling segmentation as a distribution rather than a deterministic output, this approach accounts for variability between plausible segmentations. However, the modest performance gains observed in our study suggest that architectural refinements alone are insufficient to overcome the limitations imposed by the underlying imaging modality.

### Super-resolution as a hypothesis-generating tool

SR techniques are increasingly explored in medical imaging to enhance spatial detail or support downstream analytical tasks.^12^^,^^13^ In the present work, SR-CT images should be regarded as computational hypotheses rather than anatomically faithful reconstructions. Although SR enriches image texture and spatial granularity, it cannot recreate microstructural information that is fundamentally absent from the original acquisition.

Consistent with this limitation, segmentation models trained on micro-CT identified calcified structures in selected SR-CT images, but performance remained heterogeneous and required expert interpretation. This variability reflects a persistent resolution gap that SR alone cannot overcome and highlights the need to evaluate SR based on task-specific utility rather than visual appearance.

### Emerging perspectives for translational plaque characterization

Future progress in noninvasive plaque characterization will likely require a convergence of complementary strategies. First, histology-informed learning approaches may provide stronger supervision by directly linking imaging features to biological ground truth.^14^ Second, multimodal imaging approaches integrating CT with magnetic resonance imaging or other functional imaging techniques may enhance tissue characterization beyond the capabilities of CT alone. Third, advances in CT hardware including high-resolution and photon-counting CT offer improved spatial resolution, spectral information, and contrast-to-noise ratios compared with conventional CT.^15^ Although still far from micro-CT resolution, these technologies may reduce the domain gap sufficiently to make computational transfer more effective. The framework proposed in this study provides a means to objectively assess the contribution of these developments.

From a clinical perspective, reliable plaque characterization could ultimately support lesion-specific treatment planning, particularly in heavily calcified femoropopliteal disease where device-artery interaction is critical. However, our findings caution that such applications remain aspirational at present and require careful validation.

### Study limitations

This study is limited by its small sample size and ex vivo design. Residual misregistration between micro-CT and histology cannot be excluded, and Dice scores should be interpreted as indicators of feasibility rather than performance benchmarks. Importantly, the study was not designed to demonstrate clinical applicability, but to delineate current boundaries and inform future research directions.

## Conclusions

SR enables exploratory assessment of plaque-related information transfer from micro-CT to clinical CT but does not overcome the fundamental resolution gap. By explicitly defining these limitations, this study provides a realistic framework for evaluating future imaging and computational approaches before clinical translation.

## Funding

None.

## Disclosures

None.
